# Exploring the Evolution of Breast Cancer Imaging: A Review of Conventional and Emerging Modalities

**DOI:** 10.7759/cureus.82762

**Published:** 2025-04-22

**Authors:** Maria Gabriela Cerdas, Jana Farhat, Sara I Elshafie, Faina Mariyam, Lina James, Arifa K Qureshi, Monica Potru, Paerhati Paliwei, Megha R Joshi, Godwin Abraham, Humza F Siddiqui

**Affiliations:** 1 Medicine, Universidad de Ciencias Médicas (UCIMED), San Jose, CRI; 2 Diagnostic Radiology, Faculty of Medicine, Lebanese University, Beirut, LBN; 3 Internal Medicine, Faculty of Medicine, University of Khartoum, Jeddah, SAU; 4 Internal Medicine, Kasturba Medical College, Manipal, Kozhikode, IND; 5 Medicine, Perundurai Medical College, Perundurai, IND; 6 Obstetrics and Gynecology, Buckinghamshire Healthcare NHS Trust, Aylesbury, GBR; 7 Radiology, Dr. Rajendra Gode Medical College, Amravati, IND; 8 Medicine and Surgery, Università Cattolica del Sacro Cuore, Rome, ITA; 9 Gastroenterology, Boston Children's Hospital, Boston, USA; 10 Oncology, Midland Metropolitan University Hospital, Smethwick, GBR; 11 Internal Medicine, Jinnah Postgraduate Medical Centre, Karachi, PAK

**Keywords:** breast cancer, breast ultrasound, contrast-enhanced mammogram, contrast-enhanced ultrasound (ceus), magnetic resonance imaging breast, mammography, molecular breast imaging, photoacoustic imaging, positron emission tomography computed tomography

## Abstract

Breast cancer (BC) is one of the leading causes of malignancy among women, and its prevalence is exponentially rising globally. Early and accurate imaging is critical for early detection, diagnosis, and treatment planning. This comprehensive review explores the current status of BC imaging, from the conventional methods such as mammography, ultrasound (US) and magnetic resonance imaging (MRI) to more advanced techniques including contrast-enhanced imaging, tomosynthesis, and molecular breast imaging (MBI). Conventional imaging remains the foundation for screening, as mammography is the most widely preferred modality. US and MRI are usually employed in dense breasts in highly suspicious cases that are not detected on a mammogram. However, the limitations posed by these traditional techniques can be curtailed using advanced modalities to enhance diagnostic accuracy. These emerging techniques provide faster and earlier detection of malignancy, particularly in high-risk patients, and substantially reduce the burden of missed cases. Emerging technologies, including photoacoustic imaging (PAI) and contrast-enhanced ultrasound (CEUS), show promising potential in visualizing microvascular structures and enhancing diagnostic accuracy. Additionally, artificial intelligence (AI) is revolutionizing BC imaging across all modalities by optimizing interpretation, enhancing sensitivity, and enabling personalized risk assessment. Although technological innovation continues to improve imaging quality and diagnostic precision, challenges such as cost, accessibility, overdiagnosis, and disparities in care remain a concern. Moving forward, a collaborative multimodal strategy that incorporates personalized imaging protocols and equitable access will be crucial for improving BC screening and management. The future of breast imaging lies not in replacing existing modalities but in developing a system where each technology complements the other, leading to earlier detection, more effective treatment, and enhanced outcomes.

## Introduction and background

Breast cancer (BC) is a serious health concern with an exponentially rising prevalence with an annual upward trend of 1%, posing an enormous burden on the healthcare system globally. The annual incidence of BC reported in 2020 among females was around 2.3 million worldwide [1]. Some of the highest incidence rates were observed in the United Kingdom (UK) and the United States of America (USA), with 194.4 and 131.8 per 100,000 person-years, respectively [2,3]. The annual incidence is increasing at a steeper rate of 1.4% among women aged below 50 as compared to 0.7% among women aged above 50 in the Caucasian population. Thailand has the highest prevalence of BC in comparison to the other Southeast Asian counterparts [1,3]. Much of this can be attributed to the availability of advanced diagnostic imaging and clinical protocols in the first-world countries. Significant lifestyle differences also play a critical role, with higher risks linked to nulliparity, early menarche, late menopause, obesity, exposure to radiation, smoking, and alcohol consumption [4]. Early BC detection has been linked to better outcomes primarily due to better management and subsequent cure rates [5]. Disparities in access to and quality of healthcare, lack of patient literacy, untrained healthcare staff, and public unawareness are some of the important challenges in BC detection observed primarily in developing countries and the underserved areas of the developed world [6].

**Figure 1:**
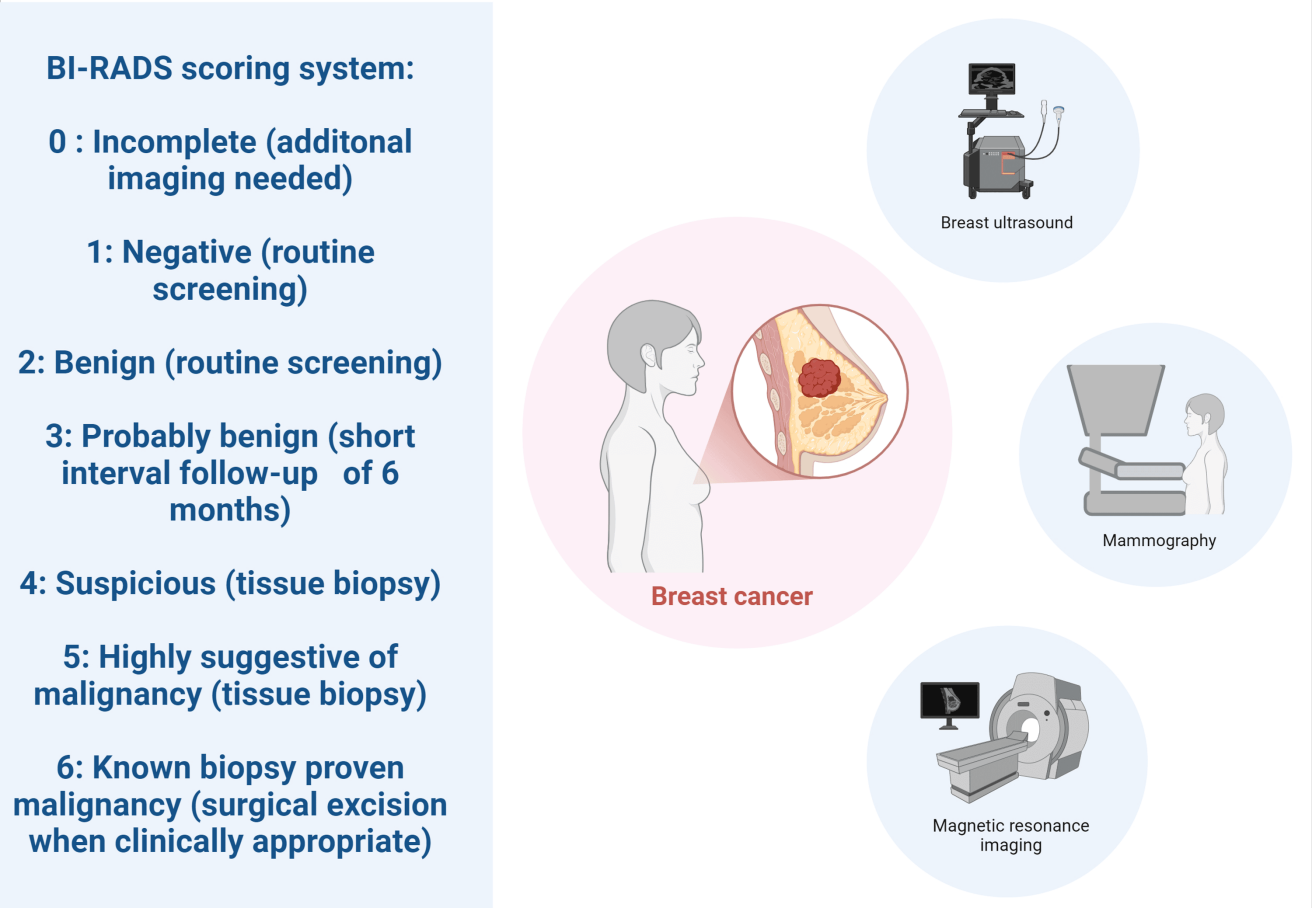
Overview of role of radiological imaging in screening and detection of breast cancer BI-RADS: Breast imaging-reporting and data system The figure has been made by Humza Siddiqui using biorender.com

All women of reproductive age are advised to perform self-examination of their breasts or receive regular examinations from healthcare providers to identify any suspicious abnormalities or lumps. Various traditional and unconventional imaging modalities are currently in use for BC screening and detection. Mammography and ultrasound are two of the most widely used preliminary imaging techniques to visualize breast abnormalities [7]. The United States Preventive Services Task Force (USPSTF) currently recommends that every woman between the ages of 40 and 74 with average risk of BC should be screened every two years via mammography [8]. The American College of Radiology (ACR) advocates the American Cancer Society recommendation of a yearly mammogram for women aged 40 to 54 for early BC detection. High-risk individuals with first-degree relatives diagnosed with BC and known BRCA 1 & 2gene mutations are generally recommended to start earlier screening at the age of 30. Breast magnetic resonance imaging (MRI) is occasionally used as an additional imaging modality in some cases, although women with a lifetime risk of less than 15% are advised against receiving an MRI [9]. The breast imaging-reporting and data system (BI-RADS) is a six-point standardized scoring system developed to provide uniform reporting of breast findings, decrease ambiguity between radiologists and referring physicians, and provide guidelines regarding the next steps of management. Patients receiving a score of 4 and above generally undergo an image-guided biopsy to further classify benign and malignant lesions (Figure 1) [10].

A recent study reported that mammograms can miss about 24% of malignancies on screening, with a mean age of 61 years at diagnosis [11]. There is a potential need for imaging modalities that can detect breast malignancies at an earlier stage with improved accuracy, faster time, and low operator dependency. The imaging should preferably provide detailed analysis to help devise individualized management strategies to enhance patient outcomes. Several modalities, including molecular breast imaging (MBI), positron emission tomography (PET) scan, contrast-enhanced ultrasound (CEUS), and mammography, have emerged recently that provide comparable or superior results [12,13]. However, it is essential to weigh potential risks and costs to implement these avenues in the clinical setting. In recent times, artificial intelligence (AI) has revolutionized medical imaging, promising standardization of diagnostic criteria, lower costs, improved accuracy, and higher accessibility [14]. This narrative review article aims to outline and highlight the advantages and limitations of conventional and emerging imaging modalities to screen and diagnose BC to aid radiologists and clinicians in formulating superior screening and diagnostic guidelines for the future to curtail the burden of breast malignancy.

## Review

Conventional imaging modalities

Ultrasound (US)

Breast US is useful in identifying masses detected on palpation and differentiating simple and complex cystic lesions. In BC screening, it is applicable in younger women as the breast tissue is predominantly dense and glandular [15,16]. This is important as higher breast density has been shown to be a risk factor for BC [17]. Additionally, US can be used to detect occult BC in clinically symptomatic patients with unremarkable mammography and to confirm the findings of abnormal mammography [18,19].

Ultrasonographic features can help differentiate between benign and malignant lesions. The sonographic appearance of benign breast masses usually includes an oval shape, a parallel orientation that is wider than tall, and a well-circumscribed margin. When comparing the histopathologic features of malignant BCs to their sonographic appearance, highly aggressive masses present as moderately heterogeneous, hypoechoic with an abrupt interface and have no posterior acoustic shadowing. Whereas less aggressive lesions tend to show spiculated margins, a markedly hypoechoic appearance with hyperechoic halos and posterior acoustic shadowing [20]. Li et al. concluded that hypoechoic areas and hypoechoic solid masses were the most common presentation of ductal carcinoma in situ (DCIS) [21]. This is further corroborated by the study of Scoggins et al. that showed that DCIS mostly presents on sonography as an irregular hypoechoic mass with indistinct margins and normal posterior features. Calcifications, architectural distortion (AD), and ductal abnormalities occur rarely [22]. Fibroadenoma, the most common benign tumor of the breast in younger women, appears as an oval, parallel, well-circumscribed, uniformly hypoechoic mass with echogenic thin fibrous internal septations and variable posterior features depending on its composition on US [23]. Other examples of benign breast lesions detected by US include simple cysts, lipomas, and masses made of glandular and fibrous tissue [24]. The systematic review conducted by Sood et al. showed that the US had an overall sensitivity and specificity of 80.1% (95% confidence interval (CI), 72.2% to 86.3%) and 88.4% (95% CI, 79.8% to 93.6%), respectively [25]. In addition, Yang et al. concluded that a supplemental breast screening by US could detect 96% (95% CI: 82 to 99%) of occult BCs missed by mammography [26]. US has a number of advantages, including wide availability, noninvasive nature, and being free of ionizing radiation exposure, making it suitable for repeated examination and providing real-time imaging and guided biopsies. However, it has a few limitations, including high operator dependency, the need for high examiner skills, and the ability to detect small lesions and early microcalcifications [23].

Mammography

Mammography is a low-dose X-ray imaging technique specifically designed to visualize breast tissue and detect abnormalities. It is considered the gold standard for BC screening, particularly for women aged 40 and older, as recommended by the ACR and the United States Preventive Services Task Force (USPSTF) guidelines. Mammograms can identify key features that differentiate between benign from malignant lesions. Benign lesions usually present as well-defined round masses, whereas malignant lesions often present as spiculated masses or clustered microcalcifications [10,11]. Studies indicate high specificity (85-90%) and moderate sensitivity (68-75%), with sensitivity decreasing in women with dense breast tissue. A study showed that the overall validity estimates of pooled sensitivity per-patient were 0.82 (95% CI: 0.76-0.87), and pooled specificities for the detection of BC were 0.84 (95% CI: 0.73-0.92). Area under the curve in receiver operator characteristics was 0.8933, and pooled sensitivity and specificity per-lesion was 76% (95% CI: 0.62-0.86) and 82% (95% CI: 0.66-0.9 [27]. Another study conducted among Japanese women aged 40-49 years showed that the sensitivity and specificity of modern film mammography, performed biennially, were 71.7% and 92.6%, respectively. The secondary analysis depicted the sensitivity of mammography was 44.1% for women with dense breasts and 34.8% for women with nondense breasts [28]. A study conducted in Denmark delineated a strong correlation between the density and texture. Sensitivity was as high as about 80% among women with a BI-RADS density score of 1 and mammography texture resemblance (MTR) markers of 1 or 2. Sensitivity was notably lower than 67% among women with a BI-RADS density score of 2 and MTR marker of 4, which further decreased among patients with a BI-RADS score of 3 and 4. Specificity was 97-99% in all subgroups [29]. In another study, mammography had a sensitivity of 80% (n = 33/41). The NPVs were found to be 60% (n = 12/20, p = 0.03), and the false-positive rate was 42% (n = 9/21). Mammography had the highest false-negative rate, missing 19% (n = 8/41) of malignant lesions [30]. A meta-analysis compared mammography alone with mammography in combination with US. The number of false-negative cases dropped from 23% in mammography alone to only 9% with the addition of ultrasonography. The combination of US and mammography was able to detect three more cancer cases per 1000 women among patients with dense breasts [31].

The major benefit of mammography screening is the reduction of BC-related deaths. Relative reductions vary from about 15% to 25% in randomized trials to more recent estimates of 13% to 17% in meta-analyses of observational studies. According to the UK population data, two to three BC-related deaths are prevented for every 1,000 women aged 50 years and above who received biennial mammography screening for two decades. However, all-cause mortality is unchanged. Based on recent estimates from the United States, the relative amount of overdiagnosis, including DCIS and invasive cancer, is 31%. This results in 15 women overdiagnosed for every 1,000 women invited to biennial mammography screening for 20 years from age 50 [32]. The American College of Surgeons’ (ACS) systematic review reported that screening mammography was associated with a decreased risk of BC mortality in randomized controlled trials (relative risk (RR): 0.80-0.82); in cohort studies (RR, 0.75; 95% CI, 0.69-0.81); and in modeling studies (median RR, 0.85; ranging from 0.77 to 0.93) [33]. The USPSTF systematic review concluded a reduced risk of advanced BC (stage IIB or greater) with screening mammography in women 50 years and older (RR, 0.62; 95% CI, 0.46-0.83) [34].

In mammography, an asymmetry is an area of increased density in one breast when compared to the corresponding area in the opposite breast. Most asymmetries are benign or caused by summation artifacts because of typical breast tissue superimposition during mammography, but an asymmetry can indicate BC. Detection and diagnosis of asymmetries are challenging because they are often subtle and appear similar to typical fibroglandular tissue in the breast. During screening mammography, asymmetries discovered that are not summation artifacts require further evaluation, such as diagnostic mammography, sonography, breast magnetic resonance imaging, or biopsy [32]. Hyperechogenicity is a sign classically reported to be in favor of a benign lesion and can be observed in many types of benign breast lesions such as hamartoma, lipoma, angiolipoma, hemangioma, hematoma, fat necrosis, fibrosis, and galactocele, among others. However, some rare malignant breast lesions can also present a hyperechoic appearance. Most of these hyperechoic malignant lesions present other characteristics that are more typically suggestive of malignancy, such as posterior shadowing, a more vertical axis, or irregular margins that help to guide the diagnosis [35]. Another study concluded that, ultimately, 344 patients with 346 AD lesions were enrolled in the study (mean age: 47.40 ± 10.07 years; range, 19-84 years). A total of 28 lesions were malignant, and 118 were nonmalignant. Palpable AD on mammography was more likely to indicate malignancy than nonpalpable AD (83.43% vs. 49.15%, p < 0.001). AD associated with other mammographic findings was more likely to be malignant than pure AD (73.58% vs. 59.36%, p = 0.005). US correlates were observed in 345 of these 346 AD lesions. Among these US correlates, 63 (18.26%, 63/345) were detected by "second look" US. For the US correlates, the mammographic ADs that appeared as non-masslike hypoechoic areas and masses on US were more likely to be malignant than those that appeared as other abnormalities (p < 0.001) [36].

Magnetic Resonance Imaging (MRI)

MRI is a highly effective imaging technique that has been employed in the screening of BC in high-risk patients. MRI is known for its high sensitivity in detecting BC often missed on mammography and US [37]. A study by Zhang et al. demonstrated that MRI outperformed mammography in sensitivity for BC screening (92% vs. 57.7%). Meanwhile, specificity (92.5% for MRI vs. 99.1% for mammography), positive predictive value (PPV) (14.2% for MRI vs. 14.6% for mammography), and negative predictive value (NPV) (99.9% for both) were almost similar between the two modalities [38]. Furthermore, a study by Pereira et al. evaluated the effectiveness of mammography, ultrasonography, and MRI in diagnosing BC. MRI showed the highest sensitivity (100%) and NPV (100%) but had low specificity (50%). Mammography had moderate sensitivity (56.2%) and high specificity (87.5%), while US had the lowest accuracy (46.9%) with poor specificity (18.8%) [39]. Additionally, a study by Aristokli et al. found that MRI had the highest sensitivity for detecting BC (94.6%), followed by US (67.2%) and mammography (54.5%) [40].

MRI helps minimize unnecessary procedures such as re-excisions and mastectomies by detecting occult BC. It is also important for monitoring the response to neoadjuvant chemotherapy and evaluating residual tumors, which might potentially be misinterpreted as post-treatment fibrosis on other modalities. MRI has also been increasingly employed in screening high-risk groups as it can identify node-negative cancers and result in better mortality rates when utilized in conjunction with mammography. It is also useful in the evaluation of occult cancers, metastatic axillary carcinoma, and the condition of silicone breast implants [41]. Additionally, it is also helpful in the diagnosis of inflammatory BC and the investigation of suspicious nipple discharge, especially when mammography and US are inconclusive. MRI is recommended in patients with benign biopsies and highly suspicious clinical signs indicating underlying disease to rule out malignancy [42].

Breast MRI is significantly more sensitive than mammography and US, particularly for high-risk women, as it can detect smaller cancers and reduce advanced-stage diagnoses. There is evidence that initiating MRI screening between the ages of 30 and 35, with the addition of mammography at age 40, reduces BC mortality among carriers of genetic mutations. MRI is also highly valuable in cases of women with dense breasts or a history of BC [43]. Contrast-enhanced T1-weighted MRI is especially effective for BC detection, outperforming other imaging methods in sensitivity. It is commonly used for preoperative evaluation, staging, follow-up treatment responses, and differentiating between scars and recurrences [44]. A study by Bae et al. found improved overall survival in women receiving both MRI and mammography compared to mammography only [45]. Furthermore, a study by Saadatmand et al. showed that MRI screening for BRCA mutation carriers or women with familial risk led to a lower incidence of distant metastases (8% in the MRI group vs. 23% in controls), and a higher 10-year metastasis-free survival rate (90% vs. 77%) [46]. Abbreviated MRI (AB-MRI) protocols decrease scan times while retaining diagnostic accuracy. It provides a quicker and less expensive alternative and captures 3D high-resolution images in approximately three minutes with diagnostic accuracy comparable to a full protocol MRI, therefore increasing access and reducing costs [47]. Ultrafast MRI techniques further reduce scan times while preserving dynamic contrast data [48].

Breast MRI is highly effective but has limitations such as false negatives and positives, which can lead to unnecessary biopsies and increased psychological distress for high-risk individuals. While MRI detects cancers at an early stage, high cost and limited availability of MRI are still significant barriers [45]. Concerns over gadolinium-based contrast agents have raised concerns regarding the potential risk of nephrogenic systemic fibrosis and gadolinium deposition. Efforts have been made to reduce the amount of contrast and scan time. Alternative techniques like unenhanced MRI and diffusion MRI are under development to expand screening options to intermediate and low-risk women. These approaches aim to balance accessibility, safety, and effectiveness while maintaining diagnostic performance [44].

Emerging imaging modalities

Positron Emission Tomography (PET) and Molecular Breast Imaging (MBI)

Different types of MBI use unique radiotracers and imaging methods to visualize molecular activity in breast tissue [49,50]. In the past couple of years, MBI systems have employed small opposing cadmium zinc telluride detectors in a dual-head configuration. This allows the breast to be sandwiched between two detectors, providing better energy resolutions and improved sensitivity to detect breast tumors of size less than 10 mm and tumors located in the upper inner quadrant. A study proves that four-fifths of invasive cancers detected by MBI were nodal negative, suggesting early detection. However, MBI was also able to detect two large node-positive occult cancers, missed on mammography [51]. MBI was also able to detect mammographically occult cancers with positive nodes, suggesting the benefits of using MBI to detect cancers that are masked in dense breast tissues. A screening trial conducted on women with dense breast, cancer detection rate of 3.2 per 1000 was achieved by mammography alone (95% CI: 1.1, 9.3), but when mammography was combined with MBI, a rate of 10.7 per 1000 (95% CI: 5.8, 19.6) was achieved (p = .016 vs mammography alone) [49].

PET-computed tomography (CT) utilizes glucose analogues such as fluorodeoxyglucose (FDG) to determine a tumor’s glucose metabolism and glycolytic activity and thus determine whether a tumor is potentially benign or malignant [52]. Precise staging for BC is crucial for curating the most correct management plan and improving prognosis. PET-CT, as an imaging technique, is not recommended for the diagnosis of primary breast malignancy due to its low sensitivity, but rather, it is used for systemic staging of breast malignancies. FDG PET-CT has been recognized to identify extra-axillary lymph nodal disease and undetected distant metastases in advanced BC, which have been missed by conventional imaging modalities like full body CT and bone imaging [53]. 

A meta-analysis comparing a whole-body PET/PET-CT with conventional imaging modalities including CT and MRI for distance metastasis staging in BC showed that across six studies (609 patients), the sensitivity for whole body PET-CT was 0.99 (95% CI: 0.88-1.00) compared to 0.57 (95% CI: 0.37-0.74) for conventional imaging. The specificity was 0.95 (95% CI: 0.89-0.98) for PET-CT compared to 0.88 (95% CI: 0.78-0.94). The positive likelihood ratio was 21.1 (95% CI: 8.2-55.5) for PET-CT compared to 4.8 (95% CI: 2.8-8.2) for conventional imaging. The study concluded that whole-body PET/PET-CT had excellent diagnostic performance for distant metastasis staging in comparison to conventional imaging modalities. The authors concluded that whole-body PET/PET-CT had a higher accuracy than conventional imaging procedures for the detection of distant metastases [54]. FDG PET-CT has also been studied for the use in disease restaging and BC recurrence. A retrospective study done in 2023 evaluated the diagnostic accuracy of routine surveillance using FDG PET-CT in the detection of clinically unsuspected recurrent BC following primary curative surgery. In this study, 1681 patients with BC who underwent curative surgery were sent for surveillance FDG PET/CT scans. Out of 2121 scans done, 105 (5.0%) showed positive findings. The use of FDG PET-CT as surveillance showed good diagnostic performance in the detection of recurrent BC, with a sensitivity of 100%, specificity of 98.5%, positive predictive value of 70.5%, and accuracy of 98.5% [55]. A study done on 748 patients across eight PET-CT studies proved sensitivity and specificity of 0.96 (95% CI: 0.90-0.98) and 0.95 (95% CI: 0.92-0.97) and for conventional imaging was 0.56 (95% CI: 0.38-0.74) and 0.91 (95% CI: 0.78-0.97) [56].

Although PET-CT is an emerging imaging technique with many benefits, some limitations need to be highlighted. According to the 2022 National Comprehensive Cancer Network (NCCN) guidelines, PET/CT imaging is only recommended for patients with stage III disease and above, when standard staging techniques provide unclear results or when patients suffer from contrast allergy [57]. Due to its high false-negative rate in detecting smaller lesions (<1 cm), the NCCN proposes that PET/CT should not be used for BC below stage III [58]. As a result, the PET-CT modality is more useful for the prognosis of advanced and metastatic disease rather than the diagnosis of primary breast malignancy and BC screening. PET-CT has a higher cumulative radiation dose exposure in comparison to PET-MRI and other nonradiation imaging modalities. Martin et al. reported that the estimated mean effective dose for whole-body PET-CT is 17.6 ± 8.7 mSv, in comparison to 3.6 ± 1.4 mSv for PET/MRI. These results show a dose reduction of 83.2% with PET-MRI when compared with full-dose PET-CT imaging [59]. Although FDG PET-CT can help recognize lesions with high glycolytic activity, inflammatory and infectious lesions might have increased uptake as well, giving false-positive results [52]. Immunotherapy-related adverse effects can cause uptake of FDG due to inflammatory changes from immunotherapy drugs such as rituximab, complicating disease monitoring [60]. 

Imaging biomarkers come into great use by overcoming these limitations, offering to detect distant metastasis in a noninvasive setting. Another method of MBI is near-infrared fluorescence (NIF), which uses dyes like methylene blue to detect BC intraoperatively [61]. This method allows surgeons to distinguish normal tissues from malignant tissues, helping in preserving structures while maintaining clean resection of the margin. The PET and MBI method of screening potentially sounds promising. However, high cost and exposure to high-dose radiation remain a big hurdle to overcoming the benefits. A recent trial conducted with a dose of 240 Mbq Tc99m sestamibi yielded similar results to 740 Mbq dose. The study noted that only 80% of cancers detected by MBI were invasive, suggesting overdiagnosis [52]. False-positive results are also observed in benign conditions like fibroadenomas, papillomas, and fat necrosis [62]. MBI showed inadequate results while detecting lesions closer to the chest wall and masses smaller than 4 mm [63].

Tomosynthesis

The concept of digital breast tomosynthesis (DBT) or 3D-mammography in breast imaging comprises the addition of a third dimension of depth into the traditional two-dimensional (2D) mammograms or full-field digital mammography (FFDM) by acquiring multiple low-dose projections of the breast using an Xray tube rotating in an arc, which can later be reconstructed and assessed using computer algorithms [64]. This reduces tissue superimposition and anatomical noise. Since its approval by the U.S. Food and Drug Administration (FDA) in 2011, several studies have been performed to compare and integrate DBT with routine screening mammograms [65].

DBT showed improved overall and invasive cancer detection rates (OR 1.45 and 1.36, p < 0.02) [66]. It also showed a superior correlation to pathological specimens when compared to FFDM in delineating lesion size, especially for dense breasts (p < 0.001) [67]. The biological profiles of cancers detected by DBM showed that they were most likely to involve dense breast parenchyma (p = 0.007), smaller size (≤2 cm, p = 0.027), and luminal A-like subtype with low Ki-67 expression (p = 0.008) [68]. The Oslo Tomosynthesis Screening Trial showed that the addition of DBT to full-field digital mammography (FFDM) improved both sensitivity and specificity (54.1 vs. 70.5, p = 0.001, and 94.2% vs. 95%, p < 0.001) at the cost of increasing radiation dose [69]. The screening with tomosynthesis or standard mammography (STORM) trial also showed that there were reduced recall rates with integrated FFDM and DBT [70]. One prospective population-based study performed in Sweden showed that DBT improved sensitivity but reduced specificity compared to FFDM alone (97.2%, 95% CI: 97.0-97.5 vs 98.1%, 95% CI: 97.9-98.3) [71]. A study comparing FFDM, DBT, and breast MRI demonstrated the superiority of DBT over FFDM and MRI [72]. Additionally, DBT has shown superiority to FFDM in image-guided biopsies by demonstrating shorter procedure time and number of exposures (p < 0.001) [73]. Though trials have shown improved sensitivity in detecting invasive cancer, long-term follow-up is required to assess if DBT will reduce BC mortality further than FFDM alone. Additionally, no differences were observed between DBT and FFDM in interval BC detection rates among women who had previously undergone baseline screening. Most of these trials were conducted in single centers with radiologists trained in DBT; therefore, the generalizability of these findings to a larger population is questionable. Larger image sets require longer interpretation time [74].

Contrast-Enhanced Mammography (CEM)

CEM combines conventional mammography with the injection of iodinated contrast agents to improve the visualization of breast tissue, particularly highlighting areas of increased vascularity associated with malignancy. CEM concurrently generates low-energy images similar to traditional digital mammography and contrast-enhanced images, offering a dual-imaging approach that improves lesion characterization and diagnostic accuracy [75].

CEM offers a number of advantages over more conventional breast imaging techniques. While being more affordable and time-efficient, it has shown comparable efficiency to breast MRI in preoperative staging and in evaluating response to neoadjuvant chemotherapy [76]. CEM beats digital mammography and ultrasonic imaging in discriminating benign from malignant cancers, showcasing favorable specificity. Potential uses of CEM include monitoring disease progression in recently diagnosed BC patients, addressing complex diagnostic problems, and potentially serving as a screening tool for high-risk women [75,77]. Patients contraindicated for MRI can benefit from this approach [75]. Furthermore, CEM is a major substitute because of its accessibility and cost benefits, particularly in settings with limited MRI access and resources. Ghaderi et al. conducted a study on CEM, which revealed a sensitivity of 93% and a specificity of 85% in spotting aggressive breast carcinomas. CEM showed a PPV of 89%, closely matching the performance of MRI [77]. Systematic review performed by Cozzi et al., including 11049 images, revealed a sensitivity of 84% and specificity of 92% of CEM among mammography-recognized abnormal findings. The CEM sensitivity slightly improved to 95%, and the specificity considerably dropped to 78% among patients with dense breasts [78]. A meta-analysis of six studies comparing the performance of CEM and MRI in detecting suspicious breast lesions showed a high sensitivity of more than 95% and a modest specificity of 77% for both modalities [79].

CEM has certain limitations, even if it offers advantages. Particularly in patients with pre-existing renal disorders, the use of iodinated contrast agents involves a small risk of allergic reactions and contrast-induced nephropathy. Though rare, around 1-3% of people may experience significant symptoms like nausea or redness. Although CEM's radiation level is considerably higher than that of traditional mammography, it stays below allowed safety limits. However, the total radiation dosage must thus be considered among patients undergoing several imaging tests. Moreover, CEM might not replace MRI, especially in some clinical settings when MRI's improved soft-tissue resolution is crucial. Technical challenges CEM faces include variability in contrast absorption and the need for accurate timing during contrast injection to improve picture quality. Further studies are warranted to assess its long-term safety and effectiveness [80].

Contrast-Enhanced Ultrasound (CEUS)

CEUS is highly effective in classifying breast lesions by visualizing their microvascular structure, offering excellent sensitivity and specificity. Research shows that integrating BI-RADS US with CEUS provides the best diagnostic accuracy for subcentimeter breast lesions. In addition, CEUS and US elastography (USE) present reliable, noninvasive alternatives to biopsy in the evaluation of small breast lesions [81].

This emerging imaging modality enhances the imaging of the breasts by using microbubble contrast agents to highlight vascular structures and abnormalities associated with malignant lesions. It can differentiate malignant from benign breast lesions, with high sensitivity (100%) and specificity (87.5%), giving results comparable to MRI. CEUS identifies distinct perfusion phases. Malignant lesions exhibit rapid enhancement and late-phase contrast accumulation, while benign lesions show delayed uniform enhancement [82]. Advantages of CEUS over conventional US include better facility and accuracy of localization concerning the sentinel lymph nodes (SLNs). A meta-analysis of studies utilizing CUES to detect BC revealed a pooled sensitivity of 88% and a specificity of 76.17% [83]. SLN enhancement with CEUS is classified into three types: homogeneous (type I), nonhomogeneous (type II), and no enhancement (type III). Type I is associated with nonmetastatic SLNs, type II with both metastatic and nonmetastatic SLNs, and type III with metastatic SLNs, helping to differentiate between metastatic and nonmetastatic lymph nodes [84]. A systematic review and meta-analysis by Deng et al. evaluated the diagnostic efficacy of SLN detection in BC using percutaneous CEUS. The analysis included 22 studies showing a sensitivity of 0.86 and a specificity of 0.89 for SLN detection using CEUS. The diagnostic odds ratio (DOR) was 53.32, and the area under the curve (AUC) was 0.94, confirming CEUS as a highly effective, noninvasive method for SLN detection in BC patients [85]. A systematic review by Zhou et al., including 5246 breast lesions, revealed a sensitivity of 0.88 and a specificity of 0.82 in differentiating benign and malignant lesions. The CEUS also showed exceptional performance in accurately predicting the pathological response to neoadjuvant chemotherapy, with a sensitivity of 0.89 and specificity of 0.83 [86].

Furthermore, CEUS plays a critical role in assessing the response to neoadjuvant chemotherapy in BC patients. It helps evaluate tumor shrinkage and predict treatment outcomes by revealing heterogeneous enhancement patterns after treatment. Combining CEUS with molecular subtypes enhances diagnostic accuracy, providing valuable insights into prognosis and therapy effectiveness [84]. CEUS is a safe and efficient imaging option for patients unable to undergo gadolinium-based procedures, those with pacemakers, or those with claustrophobia. However, challenges include occasional false positives in benign lesions caused by cellular changes or inflammation. Additionally, the current CEUS criteria may fall short in accurately classifying malignant non-mass abnormalities [87].

Photoacoustic Imaging (PAI)

PAI is a hybrid technique that shines laser light on tissue and measures optically induced US signals. The photoacoustic (PA) system provides the advantage of achieving high spatial resolution and deep penetration that can detect lesions at depths of up to 5 cm. PAI can potentially help in the early detection and staging of BC by offering better visualization of blood vessels, as angiogenesis is a hallmark of malignancy [88]. The low-intensity non-ionizing radiation utilized in PAI can alleviate the health risk related to mammography. Since PAI and US are based on similar technology, the currently available US imaging systems can be modified to generate a combined mode PA-US imaging system. This will enhance screening protocols and will prove to be cost-effective due to the existing US infrastructure. Notably, for patients with BC, PA-US is accelerating its clinical translation in macroscopic and microscopic imaging. To define the role of PA-US in clinical practice, further research should be conducted into feature analysis and interpretation strategies [89]. A study accurately detected all nine breast tumors using PA-CT within a single breath hold of 15 seconds [90]. Nyanapathi et al. presented a new portable PA system that can visualize a 7 cm thick breast and attain a spatial resolution of 1 mm in all directions within a minute [91]. Kukacka and colleagues demonstrated markedly superior image quality produced by a handheld PA imaging system. They were able to detect lesion size, type, and grade using the images accurately [92]. Neuschler et al. analyzed 1690 patients with 1757 benign and malignant masses using PA/US and US alone. Both modes showed similar sensitivity in diagnosing the lesions. However, the specificity improved by 14.9% (p < 0.0001) using PA/US in comparison to US alone [93]. Similarly, Menezes et al. downgraded 60 breast lesions to a BI-RAD score of 3 or 2, incorrectly categorized as 4 using the PA/US system, decreasing the need for invasive biopsies [94].

Artificial Intelligence (AI)

The process for BC radiomics has four main steps, including image collection, tumor identification, feature extraction, and modeling and analysis [95]. AI can help reduce the workload in mammographic screening by replacing one of the two radiologists in a double-reading system or by categorizing exams into risk groups, reducing variability between individuals. AI accelerates diagnosis, reduces waiting times, and improves treatment efficiency, leading to better patient recovery and satisfaction. AI tools have been proposed and utilized in various types of breast imaging for tasks such as detecting and classifying breast lesions, segmenting breast lesions, assessing breast density, and evaluating BC risk. AI could enhance mammogram sensitivity for those with extremely dense breast tissue by detecting occult cancers, missed by human radiologists. Radiomic models play a significant role in BC care by predicting molecular subtypes and lymph node status that aid in personalized treatment plans. They also analyze imaging features to forecast clinical outcomes such as survival, recurrence, and treatment response, enabling tailored decision-making. Radiomics bridges the gap between imaging and genetic data (radio genomics), identifying links between imaging phenotypes and genetic profiles [96,97].

AI in breast US screening improved diagnostic accuracy (ROC AUC: 0.828 to 0.848) and reduced interpretation time by 33%. AI-assisted breast US screening maintained diagnostic accuracy (ROC AUC improved from 0.828 to 0.848) while reducing interpretation time by roughly 33% (from 3:33 to 2:24 mins per case) [98]. Recent findings indicate that integrating AI into mammography screening could lower the rate of interval cancers by nearly 20%, with reductions reaching 23% for cancers with severe outcomes [99]. AI-driven systems for breast MRI improve cancer detection by using early-phase spatial and symmetry information. This approach showed a 20% increase in sensitivity (p = 0.008) compared to traditional methods, enhancing accurate screening in abbreviated MRI protocols [100].

The responsible use, labeling, and protection of data is crucial to ensure privacy and integrity. Accountability for mistakes resulting from AI usage must be clearly defined. The monitoring and verification of AI-driven autonomy is essential to maintain ethical standards and prevent errors in clinical practice. The absence of robust evidence and regulations to back the use of AI models, along with the lack of standardized benchmarks, creates challenges in validating their performance effectively. Picture Archiving and Communication Systems (PACS) and Digital Imaging and Communications in Medicine (DICOM) have streamlined the preparation of datasets for AI-based applications, ensuring easy access and retrieval. However, the organization of these datasets, including tasks such as labeling, annotation, and segmentation, remains a significant challenge [97]. It requires skilled professionals such as clinical scientists and informaticians, making it a time-intensive and costly process. Several radiomic models lack thorough external validation, and their reproducibility across various patient populations remains questionable. Addressing these obstacles is crucial for the successful integration of AI into clinical BC care [101]. Model bias is another concern, particularly with the underreporting of critical demographic data such as race and ethnicity, which can affect the generalizability of the model [102].

Future studies should assess AI performance and AUC by exploring various AI-radiologist collaborations, including using AI as an assistive tool in interpretation and as an independent approach. Additionally, evaluating the cost-effectiveness of AI in screening processes should be considered [96]. A deep learning-based CAD (DL-CAD) tool has recently been introduced for detecting and characterizing breast lesions as either benign or malignant. The transition from traditional CAD systems to advanced AI tools like DL-CAD is expected to hold the potential to minimize false positives, enhance diagnostic accuracy, boost radiologist performance, and support clinical decision-making. Efforts toward global standardization aim to create uniform diagnostic protocols across institutions [97].

## Conclusions

BC imaging has had significant advancements, ranging from traditional methods such as mammography and ultrasound to more sophisticated modalities like MRI, PET-CT, and molecular imaging. Mammography has historically been the primary method employed for early detection, contributing to a reduction in mortality rates. Its limitations, especially in dense breast tissue, have necessitated the use of other modalities, such as US and MRI, which offer enhanced sensitivity and specificity. In resource-limited settings, issues such as radiation exposure, elevated costs, and accessibility challenges persist despite these advancements.

Advancements in AI, hybrid imaging modalities, and precision medicine methodologies are likely to shape the future trajectory of BC imaging. Emerging imaging technologies such as PET-MRI and radio genomics will provide more tailored screening and treatment strategies, while AI-driven models will enhance diagnostic precision and optimize healthcare operations. Research must focus on harmonizing technological innovation with cost-effectiveness and global accessibility. Advancements in BC imaging will significantly enhance early diagnosis, treatment outcomes, and overall patient survival via continuous advances and multidisciplinary collaboration.
